# Connecting a Western diet, palmitic acid, and enteric neuropathy

**DOI:** 10.1172/JCI205830

**Published:** 2026-06-01

**Authors:** Rexford S. Ahima

**Affiliations:** Department of Medicine, Division of Endocrinology, Diabetes and Metabolism, Johns Hopkins University School of Medicine, Baltimore, Maryland, USA.

## Abstract

The Western diet (WD) is a rich source of saturated fatty acids, especially palmitic acid (PA), which has been implicated in the pathogenesis of insulin resistance, oxidative stress, inflammation, diabetes, and multiorgan dysfunction in obesity and diabetes. In this issue of the *JCI*, a study by Balasubramaniam et al. describes mechanisms linking a WD, PA, ferroptosis (iron-dependent cell death), and loss of colonic motility. Chronic PA exposure drove ferroptosis in murine in vitro systems and human myenteric ganglia. Mice fed a WD for 12 weeks developed enteropathy and loss of colonic motility, which was reversed by adeno-associated virus–mediated (AAV-mediated) overexpression of the transcription factor NFE2L2, preventing ferroptosis and restoring redox balance to enteric neurons. The study provides critical data establishing PA-induced ferroptosis as a mediator and potential therapeutic target in enteric nervous system disorders associated with obesity.

## Introduction

**Figure 1:**
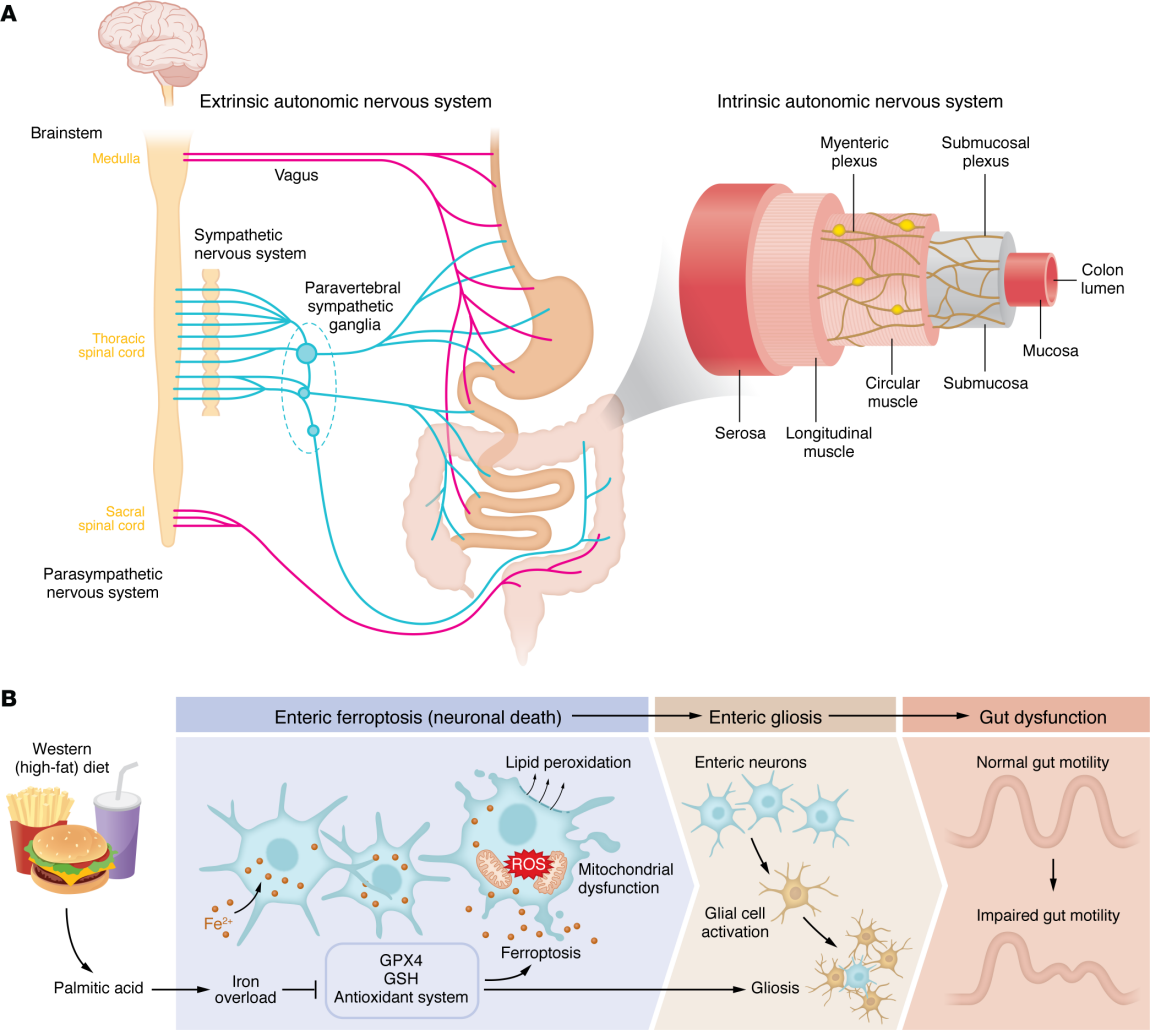
ENS dysfunction in diet-induced obesity. (**A**) Innervation of the GI tract. Schematic showing the extrinsic ANS and the intrinsic ENS. Sympathetic (blue) and parasympathetic (pink) fibers originate from the CNS and synapse with the ENS within the myenteric and submucosal plexus to regulate gut motility, secretion, vascular function, and interactions with the immune system and gut microbiota. (**B**) Balasubramaniam et al. (8) connected consumption of a Western HFD to loss of enteric neurons and gut motility. HFD consumption resulted in elevated saturated fatty acids, specifically PA, which promoted increased iron influx into enteric neurons, leading to iron overload. This excess iron, combined with PA, reduced glutathione (GSH) levels and inhibited the activity of GPX4, a critical inhibitor of lipid peroxidation. Consequently, elevated free iron levels and lipid peroxidation caused mitochondrial dysfunction and increased ROS production, enteric neuronal death (ferroptosis), and reactive gliosis, culminating in enteric neuropathy and impairment of colonic motility.

The enteric nervous system (ENS), often considered the “second brain,” is a key component of the autonomic nervous system (ANS) embedded within the gastrointestinal (GI) tract. The myenteric (Auerbach’s) plexus is localized between the longitudinal and circular muscle layers, and controls GI motility by regulating the force and rhythm of muscle contractions to move the GI contents. The submucosal (Meissner’s) plexus is situated in the submucosa and plays an integral role in the regulation of GI secretion, blood flow, and nutrient absorption (Figure 1A) (1, 2).

The ENS is composed of diverse neuronal types including sensory, motor, and interneurons, which form ganglia that function independently as well as interact with the central nervous system (CNS) to sense local signals and coordinate central inputs to control GI motility, digestion, and neuroendocrine functions (1–3). Enteric neurons interact closely with glia, interstitial cells, enteroendocrine cells, blood vessels, and gut microbes to maintain neuronal functions, mucosal integrity, and immune responses. The ENS network plays a critical role in digestive function and the pathophysiology of GI disorders (1–3).

GI symptoms, such as bloating, constipation, and motility disorders are more common in individuals with obesity and/or diabetes (4, 5). The pathophysiology of diabetic enteric neuropathy involves multiple factors: chronic hyperglycemia, autonomic and vascular dysfunction, inflammation, neurotransmitter alterations, alteration of gut microbiota, and tissue remodeling (4, 5). There is evidence for ENS damage in people with obesity, and animal models have demonstrated an association of a high-fat diet (HFD) and enteric neuropathy (reviewed in ref. 5). Adult mice fed a HFD are prone to enteric neuropathy, and perinatal mice fed a HFD have decreased ENS neurons prior to the onset of obesity (reviewed in ref. 5). Thus, it is plausible that dietary and other metabolism-related factors contribute to the pathophysiology of enteropathy and GI motility dysfunction in obesity.

Palmitic acid (PA) is the predominant dietary saturated fatty acid in diets consumed in the United States (6, 7). This so-called Western diet (WD), characterized in part by high fat intake, is a major source of PA, which is taken up the liver, adipose tissue, heart, and skeletal muscle and promotes insulin resistance and diabetes (6, 7). Plasma PA can also be produced via de novo synthesis in the liver and adipose tissue after consumption of a high-carbohydrate diet. Elevated PA levels are associated with lipotoxicity, oxidative stress, liver, muscle, and pancreatic β cell dysfunction, and neuronal death (6, 7).

In this *JCI* issue, Balasubramaniam et al. (8) tested the hypothesis that PA overload from a WD induces ferroptosis, resulting in ENS neuropathy and disruption of colonic motility (Figure 1B). Ferroptosis is a type of iron-dependent regulated cell death, distinct from apoptosis and necrosis, that is involved in the pathogenesis of neurodegenerative diseases in the CNS (9, 10). Ferroptosis is characterized by cellular accumulation of iron, lipid peroxidation, and excessive ROS, which collectively lead to loss of plasma membrane and neurodegeneration (9, 10).

## PA induces ferroptosis in murine enteric neurons

To investigate whether PA induces ferroptotic stress and cellular death, Balasubramaniam et al. chronically exposed a murine enteric neuronal IM-FEN cell line to PA (0.5 mM for 24 hours). This PA concentration is within the dose range of plasma PA levels in WD-fed animals. RNA-seq of PA-treated enteric neurons revealed a significant upregulation of transferrin receptor 1 (encoded by *Tfr1*), divalent metal transporter 1 (encoded by *Dmt1*), and major genes encoding major iron influx transporters, whereas *Slc40a1*, encoding ferroportin, which mediates cellular iron export, was downregulated. The RNA-seq results were confirmed by reverse transcription PCR (RT-PCR) showing elevated expression of *TfR1*, *DMT1*, and the proinflammatory cytokine *IL-6* and reduced expression of glutathione peroxidase 4 (*GPX4*) and *SLC40A1*. Immunoblots demonstrated increased protein levels of ferritin heavy chain 1 (FTH1), indicating excessive iron storage, and this was corroborated by immunofluorescence of FTH1 in enteric neurons. Intracellular iron overload was assessed by measuring labile Fe²^+^ using FeRhoNox-1 staining. Chronic PA exposure significantly increased cytosolic Fe²^+^ levels, and cotreatment with the ferroptosis inhibitor ferrostatin 1 (Fer-1) prevented this response (8).

In addition to increasing iron accumulation in enteric neurons, chronic exposure to PA resulted in substantial neuronal degeneration. RNA-seq analysis revealed downregulation of neuronal markers, upregulation of oxidative stress genes, and increased neuronal death, as shown by propidium iodide staining indicating plasma membrane rupture and cell death. Inhibition of ferroptosis with Fer-1 significantly reduced enteric neuronal death. Immunofluorescence of PA-treated enteric neurons showed elevated levels of the lipid peroxidizing enzyme arachidonic acid 15-lipoxygenase (ALOX15) and reduced levels of GPX4, the main antioxidant enzyme that prevents lipid peroxidation. Fer-1 treatment rescued lipid peroxidation and prevented enteric neuronal death. Together, these results demonstrate a model in which chronic PA exposure triggers cellular iron accumulation, lipid peroxidation, and neuronal death (8).

The investigators also studied the ferroptotic stress response and mitochondrial dysfunction in PA-treated murine enteric neurons (Figure 1B). Gene expression profiling revealed changes in heat shock proteins and in genes modulating cellular stress, ROS production, and disruption of mitochondrial function. Histology showed that PA increased MitoSOX Red staining indicating elevated mitochondrial ROS levels and MitoFerroGreen staining consistent with mitochondrial labile Fe^2+^. These changes were reversed by Fer-1 treatment. MitoBrilliant 646 imaging showed fragmented mitochondrial networks, and expression of mitoferrin 2 (MFRN2) was increased, suggesting enhanced mitochondrial iron accumulation (8).

To distinguish between acute (20 minutes) versus chronic (24 hours) effects of PA exposure, the investigators examined electrical field stimulation–evoked (EFS-evoked), frequency-dependent Ca²^+^ responses in Fluo-4–loaded enteric neurons cells. Chronic exposure to 0.5 mM PA (24 hours) markedly suppressed EFS-evoked Ca²^+^responses and caused cell death; and again, these effects were prevented by Fer-1 pretreatment. In contrast, acute PA exposure elicited divergent dose-dependent EFS responses. PA 0.1 mM strongly inhibited EFS-evoked Ca²^+^ responses, while PA 0.5 mM enhanced EFS responses. The EFS response to chronic PA was blocked by tetrodotoxin (TTX), whereas the acute PA EFS responses were insensitive to TTX, implicating voltage-gated sodium channels (Nav) in the chronic but not acute response to PA. Unlike chronic PA exposure, ferroptosis inhibition via Fer-1 did not affect the acute EFS response. Mechanisms underlying the differences in acute versus chronic responses to PA are unknown. Palmitoylation involving addition of PA to cysteine residues is a reversible posttranslational lipid modification that regulates protein functions, e.g., the trafficking, localization, and electrical activity of Nav subtypes in various cells (11, 12). Palmitoylation of Nav1.6 at the Cys1978 residue increases neuronal current amplitude and action potential generation (12). Further studies are needed to elucidate whether the duration of PA exerts differential effects on Nav1.6 and enteric neuronal activity.

## WD suppresses phosphorylated NFE2L2’s antioxidant effect and colonic motility

Nuclear factor erythroid-derived 2–like 2 (NFE2L2, also known as NRF2), a transcription factor that controls antioxidant and lipid-peroxide detoxification pathways, restrains ferroptotic susceptibility (13). Balasubramaniam et al. observed that chronic PA exposure significantly reduced nuclear phosphorylated NFE2L2 (p-NFE2L2) expression in enteric neurons, and cotreatment with Fer-1 restored p-NFE2L2 levels (8). This suggested that NFE2L2 insufficiency may contribute to the deleterious effects of PA and a WD.

They therefore investigated the in vivo function of NFE2L2 via AAV-MaCPNS2 capsid injection, which efficiently transduces peripheral neurons, including enteric neurons. After 12 weeks, there was a significant increase in *Nfe2l2* mRNA expression and immunofluorescence staining in enteric neurons of AAV-NFE2L2–treated mice compared with AAV-EGFP controls. Mice fed a WD showed significantly delayed colonic motility as measured by bead expulsion time, and overexpression of NFE2L2 in enteric neurons prevented the delayed colonic motility.

Next, Balasubramaniam et al. examined the effect of in vivo enteric neuronal expression of NFE2L2 on ferroptotic markers. After 12 weeks, the WD-fed mice gained more weight than did mice fed a control diet (CD). Immunofluorescence analysis of colonic tissue revealed that mice fed a WD had increased expression of TfR1 and FTH1 in myenteric ganglia compared with those fed a CD. AAV-NFE2L2 overexpression markedly reduced TfR1 and FTH1 levels, indicating a role of NFE2L2 in regulating neuronal iron metabolism. This was associated with suppression of neuronal and ROS markers. The ferroptotic markers were colocalized specifically in neurons within the ENS ganglia, which was associated with markers of oxidative stress (8). Together, the findings support a role for NFE2L2 in regulating ferroptosis in enteric neurons to maintain colonic function.

## PA induces ferroptotic responses in human ENS cells

To determine whether PA-induced ferroptosis occurs in the human ENS, experiments were conducted in freshly isolated myenteric ganglia from surgical intestinal specimens from patients undergoing colon resection (8). This in vitro human ENS model was treated with PA (0.5 mM) for 24 hours and evaluated for ferroptosis using high-resolution confocal imaging. PA exposure resulted in neuronal death compared with vehicle-treated controls. PA increased TfR1 expression in human enteric neurons; other ferroptosis inducers such as ferric ammonium citrate and LPS also caused TfR1 upregulation in human enteric neurons (8).

PA also increased FTH1 in enteric neurons, and the signal was morphologically localized within the somatic cytoplasm and proximal neurites. Notably, FTH1 staining was significantly elevated in non-neuronal regions of the ganglia, including glial-like territories not colabeled with the neuronal marker. This finding suggests that FTH1 is a marker of ferroptotic stress in both neurons and glia. To evaluate the effect of PA on glial stress responses, the investigators analyzed the expression of glial fibrillary acidic protein (GFAP), which would be expected to be absent or minimally expressed in healthy human enteric glia but strongly induced in response to inflammation. Following PA treatment, GFAP was increased, suggesting activation of gliosis in response to PA-induced cellular stress (Figure 1B). Together, these findings demonstrate that ferroptotic injury in the human ENS results in neuronal disruption and glial reactivity.

## Conclusions and future directions

This study provides several important insights into the pathophysiology of enteric neuropathy: (a) identification of chronic PA and WD exposure as potent inducers of ferroptosis and enteric neurodegeneration in murine and human ENS; (b) demonstration in mice that NFE2L2 activation restores antioxidant balance, suppresses ferroptotic neuronal death, and improves colonic motility under stress caused by a WD; (c) demonstration that both the mouse and human ENSs show ferroptotic responses to PA, including iron overload, neuronal death, and glial activation.

However, there are limitations requiring further investigation. The ferroptotic changes and functional assays in this study were limited to the colon and may vary in other segments of the GI tract. The in vivo complexity of how WD affects other lipid species, metabolic mediators, the microbiota, immune modulation, and GI mucosal interactions remains to be studied. The current study also did not examine the functional enteric neuron-glia interactions in ferroptosis or the effect on GI motility, or how this local neuron-glia network interacts with the CNS. Moreover, it is unclear whether the mechanisms linking PA, a WD, ferroptosis, and enteropathy apply to obesity as well as diabetes. Despite these limitations, the study provides critical evidence establishing PA-induced ferroptosis as a mediator and potential therapeutic target in ENS disorders associated with abnormal GI motility.

## Conflict of interest

The author has declared that no conflict of interest exists.

## Funding support

This work is the result of NIH funding, in whole or in part, and is subject to the NIH Public Access Policy. Through acceptance of this federal funding, the NIH has been given a right to make the work publicly available in PubMed Central.

- Bloomberg Distinguished Professorship (to RSA).
- NIH grant R01DK135751 (to RSA).

## Version 1 06/01/2026

Electronic publication
